# Small-Conductance Ca^2+^-Activated K^+^ Channels 2 in the Hypothalamic Paraventricular Nucleus Precipitates Visceral Hypersensitivity Induced by Neonatal Colorectal Distension in Rats

**DOI:** 10.3389/fphar.2020.605618

**Published:** 2021-01-27

**Authors:** Ning-Ning Ji, Lei Du, Ying Wang, Ke Wu, Zi-Yang Chen, Rong Hua, Yong-Mei Zhang

**Affiliations:** ^1^ Jiangsu Province Key Laboratory of Anesthesiology, Xuzhou Medical University, Xuzhou, China; ^2^ Anesthesiology Department of the Nanjing Children’s Hospital, Nanjing, China; ^3^ Department of Anesthesiology, The First Affiliated Hospital of Nanjing Medical University, Nanjing, China; ^4^ Institute of Emergency Rescue Medicine, Xuzhou Medical University, Xuzhou, China

**Keywords:** PKA, neonatal colorectal distension, visceral hypersensitivity, rats, hypothalamic paraventricular nucleus, small-conductance Ca^2+^-activated K^+^ channel 2

## Abstract

Visceral hypersensitivity is one of the pivotal pathophysiological features of visceral pain in irritable bowel syndrome (IBS). Small-conductance Ca^2+^-activated K^+^ channel (SK) is critical for a variety of functions in the central nervous system (CNS), nonetheless, whether it is involved in the pathogenesis of visceral hypersensitivity remain elusive. In this study, we examined mechanism of SK2 in hypothalamic paraventricular nucleus (PVN) in the pathogenesis of visceral hypersensitivity induced by neonatal colorectal distension (CRD). Rats undergoing neonatal CRD presented with visceral hypersensitivity as well as downregulated membrane SK2 channel and p-PKA. Intra-PVN administration of either the membrane protein transport inhibitor dynasore or the SK2 activator 1-EBIO upregulated the expression of membrane SK2 in PVN and mitigated visceral hypersensitivity. In addition, 1-EBIO administration reversed the increase in neuronal firing rates in PVN in rats undergoing neonatal CRD. On the contrary, intra-PVN administration of either the SK2 inhibitor apamin or PKA activator 8-Br-cAMP exacerbated the visceral hypersensitivity. Taken together, these findings demonstrated that visceral hypersensitivity is related to the downregulation of membrane SK2 in PVN, which may be attributed to the activation of PKA; pharmacologic activation of SK2 alleviated visceral hypersensitivity, which brings prospect of SK2 activators as a new intervention for visceral pain.

## Introduction

Visceral hypersensitivity is one of the key pathophysiological features of irritable bowel syndrome (IBS) as well as other conditions with visceral pain (Chey et al., 2015). The mechanism underlying visceral hypersensitivity remains unclear on the grounds of absence of structural abnormalities detected in the internal organs. Our prior study demonstrated that re-exposure to CRD in adult rats having been subjected to neonatal CRD induced visceral hypersensitivity (Yu et al., 2014; Chen et al., 2015; Zhang et al., 2016b; Song et al., 2018). The hypothalamic paraventricular nucleus (PVN) integrates multiple afferents to autonomously regulate visceral sensation (Li et al., 2014). Corticotropin-releasing factor (CRF) neurons in PVN increased corticosteroids and adrenocorticotropic hormone (ACTH) levels via the hypothalamic–pituitary–adrenal (HPA) axis, which can be disrupted by stress-induced PVN neuroplasticity and HPA axis dysregulation (de Kloet et al., 2005; Van den Bergh et al., 2008; Bravo et al., 2011; Green et al., 2011; Amath et al., 2012). Our prior studies confirmed that CRD induced visceral hypersensitivity as well as enhanced excitability of CRF neurons, with both CRF protein expression in PVN and plasma cortisol levels increased (Yu et al., 2014; Chen et al., 2015). The activation of CRF neurons in PVN leads to the CRF release, resulting in the undermined analgesia (Lariviere and Melzack, 2000) and exacerbated neuropathic pain (Fu et al., 2016), thereby implying the pivotal role of CRF neurons in PVN in modulating visceral hypersensitivity. However, the mechanism underpinning the elevated excitability of CRF neurons in PVN in the pathogenesis of visceral hypersensitivity remains elusive.

SK channels, which are an important membrane ion channels regulating neuronal excitability, consist of four subtypes (SK1, SK2, SK3 and IK1channels) and are widely distributed across the CNS (Kohler et al., 1996; Faber, 2009; Adelman et al., 2012; Deignan et al., 2012; Li et al., 2017). Activation of SK2 channel is prerequisite for the control of K^+^ outflow and the consequent generation of the medium afterhyperpolarizations (mAHP) in response to the increase of the intracellular Ca^2+^ levels, with the firing rate decreased and the excitatory of neuron inhibited (Pedarzani et al., 2000; Strassmaier et al., 2005; Hammond et al., 2006; Chang et al., 2013). It is well known that abnormal neuronal excitability is one of the mechanisms of pain (Cheng et al., 2015; Fan et al., 2015) and SK channels are reportedly associated with nociception. Administration of the selective SK channel blocker UCL1848 increases neuronal responses to naturally evoked nociceptive stimuli. Conversely, administration of the selective SK channel activator 1-EBIO inhibits neuronal responses evoked by mechanical stimuli via the increased SK channel activity (Bahia et al., 2005). Administration of the selective SK2 channel blocker apamin increased excitability and enhanced excitatory synaptic transmission, as indicated by increased frequency of miniature EPSCs and action potentials, leading to hyperexcitability and pain hypersensitivity (Pagadala et al., 2013). SK channels in the amygdala mediate pain-inhibiting effects in a rat model of arthritic pain (Thompson et al., 2015). These findings on the multitude of effects associated with SK2 channel invited our exploration of the implications of SK2 channel in PVN in rat model of visceral hypersensitivity.

In this study, we examined the mechanism of SK2 channels underlying elevated excitability of CRF neurons in PVN in the pathogenesis of visceral pain. Our data suggested that visceral hypersensitivity is related to the downregulation of SK2 channel protein as well as inactivation of SK2 channel in PVN CRF neurons. Our findings might provide new molecular and neuronal insights into the precipitation of visceral hypersensitivity, thereby benefiting the medical intervention regimens with SK2 activators for visceral pain.

## Materials and Methods

### Animals

Neonatal sucking male Sprague-Dawley rats (within 8 days) were provided from the Experimental Animal Center, Xuzhou Medical University (Xuzhou, China) and kept with the maternal rats until 21 days. After weaning, the young male rats were housed in fours in standard Plexiglas cages, with ad libitum access to food and water. Rats were checked daily and weighed weekly for 2 months or until body weight of 200–250 g prior to group designation. During the testing session, rats were maintained on a standard 12 h light–dark cycle (lights on at 07:00 a.m. and off at 07:00 p.m.), with constant temperature and humidity (22°C and 50%, respectively) and ad libitum access to food and water. Animal sample sizes (6 rats per group) were determined by the expected change of the experiments and previous experience from similar studies and were sufficient for all statistical tests (alpha = 0.05; power = 0.90; one-tailed test). Statistical analyses were performed with PASS version 15.0. The experimental protocol design was based on the Replacement, Reduction and Refinement principles described 2010/63/EU law on Animal Protection Used for Scientific Experiments. All procedures were conducted in accordance with the guidelines of the National Institutes of Health’s Guide for the Care and Use of Laboratory Animals (NIH Publication No. 8023, revised 1978) and the International Association for the Study of Pain, and were approved by the Institutional Animal Care and Use Committee at Xuzhou Medical University.

### Reagents

The reagents and antibodies were as follows: rabbit anti-KCa2.2 (APC-028) polyclonal Ab (Alomone Labs, Jerusalem, Israel); mouse anti-GAPDH (AC001) mAb (Abclonal, Woburn, MA, United States); alkaline phosphatase goat anti-rabbit IgG (ZB-2308); alkaline phosphatase horse anti-mouse IgG (ZB-2310); BCA protein assay kit (P0012); sodium dodecyl sulfate (SDS)-polyacrylamide gel electrophoresis (PAGE) sample loading buffer (P0015); BCIP/NBT alkaline phosphatase color development kit (C3206). Syn-PER™ Synaptic Protein Extraction Reagent (#87793) (Thermo Fisher Scientific, Waltham, MA, United States). AAV-CRH-EYFP-WPRE-pA (AAV-CRH-EYFP) was purchased from BrainVTA (Wuhan) Co., Ltd.

### Visceral Hypersensitivity Model

Chronic visceral hyperalgesia model was established by repeated CRDs in neonatal rats. Neonatal rats were subjected to CRDs on postnatal days 8, 10, and 12, via an angioplasty balloon (20.0 mm in length and 3.0 mm in diameter) inserted into the rectum and descending colon. The balloon was distended at a pressure of 60 mmHg for 1 min prior to deflation and withdrawal, with the distention repeated daily and at an interval of 30 min. The neonatal rats were returned to the maternal rats immediately after each CRD procedure. Neonatal CRD, i.e. model rats were weaned on day 21, and separated into different cages on day 30. CRD rats were routinely raised till the postnatal 8^th^ week. By then, the adult rats underwent CRDs at 60 mmHg for 60 s, ten times at an interval of 15 s, so as to trigger visceral hyperalgesia.

### Pain Threshold

Visceral sensitivity was assessed via pain threshold. Rats were placed in Lucite cubicles (20 × 8 × 8 cm) on an elevated Plexiglas platform and allowed for habituation for 15–30 min. Graded distension was exerted by rapid inflation of a balloon inside the descending colon and rectal region to a desired pressure (20, 40, 60, or 80 mmHg) for a duration of 20 s followed by a 4-min rest. The abdominal withdrawal reflex (AWR) was scored as: 0, no behavioral response to distension; 1, slight head movement followed by immobility; 2, contraction of the abdominal muscles; 3, lifting of the abdomen; 4, body arching and lifting of the derriere. The pain threshold by distension was defined as an AWR score of 3. For accuracy, each distension procedure was in triplicate. Naïve rats did not undergo any treatment.

### Intra-Paraventricular Nucleus Microinfusion

Rats were anesthetized under 2% pentobarbital sodium (40 mg/kg) and mounted onto a David Kopf stereotaxic frame (Tujunga, CA, United States), with the cranium in a horizontal plane. With the scalp incised and holes drilled through the cranium for bilateral insertion of a microinjector needle (28 gauge) into the PVN region of the hypothalamus (A/P −1.5 mm, L/R ± 0.4 mm, D/V −7.7 mm from bregma), Dynasore (80 μM, 0.5 μl), EBIO (10 μg/0.3 μl), apamin (6.25 pmol/0.3 μl) or 8-Br-cAMP (100 mM, 0.1 μl) (Wang et al., 2012) was infused into PVN in 5 min, with the microinjector needle in place for an additional 5 min to allow for solution diffusion prior to the skin closure. 30 min thereafter, rats underwent behavioral tests or brain isolation. The site of the cannula track aiming at PVN was histologically verified for each brain, and rats with incorrect cannulation were excluded from data analysis. The virus (AAV-CRH-EYFP, AAV2/9, 2 × 10^12^ viral genome ml^−1^, 300 nl) to label PVN CRF neurons was injected with identical procedure, and brain sections were sliced for electrophysiological recording 21 days thereafter.

### HT-22 Hippocampal Neurons

Hippocampal HT22 cells were cultured in DMEM-HAMS F12, supplemented with 10% fetal bovine serum, L-glutamine (100 mM) and 1% antibiotics (penicillin, streptomycin) and incubated in humidified 5% CO_2_ atmosphere at 37°C. At 80% confluence, cells were detached with trypsin-EDTA, rinsed and sub-cultivated in new flasks for 1–2 days prior to experimentation. The cells were then incubated at 37°C with 80 μmol/L dynasore for 30 min.

### Quantitative Real-Time Reverse Transcription-Polymerase Chain Reaction

The SK2 mRNA level in PVN was determined by means of qRT-PCR. Total cellular RNA was isolated from tissue samples via Trizol Reagent (15,596–026, Invitrogen, Carlsbad, CA, United States) as per the manufacturer’s protocol. The RNA was quantified by spectrophotometry (OD 260/280). RNA was transcribed to cDNA using M-MLV Reverse Transcriptase (D263915) and dT primers. PCR amplification was performed with Taq polymerase using 40 cycles at 94°C for 30 s, 58°C for 30 s, and 72°C for 1 min. The PCR primers for SK2 were 5′-TTG​TGG​AAG​GGG​CAT​AGG​AGA-3′ (sense) and 5′-AATGGAGCAGATGA CTGGAGA-3′ (antisense), for GAPDH were 5′-TCT​CTG​CTC​CTC​CCT​GTT​C-3′ (sense) and 5′-ACA​CCG​ACC​TTC​ACC​ATC​T-3′ (antisense), which were synthesized by Invitrogen Biotech Co. Ltd. (Shanghai, China). The qRT-PCR was performed with a Rotor-Gene 3,000 real-time DNA analysis system (Corbett Research, Sydney, Australia) with real-time SYBR Green PCR technology. The reaction mixtures contained diluted cDNA, SYBR Premix Ex Taq II (2×; DRR081), 10 μM of each gene-specific primer, and nuclease-free water in a final volume of 10 μl. The cDNA results were normalized to glyceraldehyde-3-phosphate dehydrogenase (GAPDH) measured for the same sample.

### Western Blotting

With the rats sacrificed and the brain quickly isolated in the ice bath, the PVN area was transferred into individual freezing storage tubes, followed by addition of Syn-PER™ Synaptic Protein Extraction Reagent (1 ml/100 mg) containing phosphatase inhibitors and PMSF. With the tissue fully homogenized by a homogenizer on ice, samples were centrifuged at 8,000 rpm for 10 min at 4°C. Following collection of the supernatant (containing cytoplasmic protein) and further centrifugation at 12,000 rpm for 20 min at 4°C, lysis buffer was added for dissolution of the deposition (containing membrane protein). Protein concentration was measured by the BCA Protein Assay kit (P0012). 25 μg of brain tissues was added and separated by 10% SDS-PAGE gel system for electrophoresis with the PageRuler Prestained Protein Ladder (26,616, Thermo). Thereafter, the protein was transferred onto the PVDF membrane at the constant voltage of 100 V and approximately 60 min with a wet transferometer. After the protein transference, the PVDF membrane was rinsed in washing buffer for 5 min, followed by addition of 5% skim milk, at room temperature (r/t) for 2 h. Membranes were then incubated in rabbit monoclonal anti-GAPDH (1:1,000, G9545, Sigma-Aldrich, Co. LLC, MO, United States) and rabbit anti-KCa2.2 (1:1,000, APC-028, Alomone Labs) or rabbit monoclonal anti-p-PKA (1:1,000) overnight at 4°C. On the following day, after 30 minutes of rewarming, the PVDF membranes were rinsed with washing buffer for 10 min in triplicate, and were then incubated in anti-rabbit IgG with alkaline phosphatase (1:1,000; A0208, Beyotime) on the shaking bed at r/t for 2 h, with BCIP/NBT alkaline phosphatase color development kit (C3206) employed for protein coloration. ImageJ software was used for grayscale analysis.

### Electrophysiological Recording

Transverse sections of the PVN area were taken at 250–300 mm in ice-cold slicing solution (mM): NaCl, 80; KCl, 3.5; MgSO_4_, 4.5; CaCl_2_, 0.5; NaH_2_PO_4_, 1.25; sucrose, 90; NaHCO_3_, 25 and glucose, 10. Artificial cerebral spinal fluid (aCSF) (mM) consisted of NaCl, 126; KCl, 2.5; NaH_2_PO_2_, 1.2; MgSO_4_, 1.2; NaHCO_3_, 26; glucose, 10 and CaCl_2_, 2.4. All solutions were saturated with 95% O_2_ and 5% CO_2_. The slices were incubated in cutting solution at 32°C for 15 min, and were then transferred into the aCSF at r/t for at least 1 h prior to submersion in recording chamber. Whole cell patch-clamp recording was conducted in CRF neurons with a glass pipette filled with an internal solution (mM): phosphocreatine-Tris, 10; 10 HEPES, 10 EGTA, 2 ATP-Mg, 0.5 GTPNa, 115 K gluconate, 20 KCl, 1.5 MgCl_2_; with pH adjusted to 7.2 with KOH (285 mOsm). The resistance was 4–8 MΩ. Whole cell recoding: cells were maintained in the current-clamp mode at -60 mV and action potential firings in response to the injection of depolarizing current pulses were recorded with a patch-clamp amplifier (MultiClamp 700A, Axon Instruments, Union City, CA, United States). For measurement of SK currents, CRF neurons were held in voltage-clamp at a holding potential of −60 mV and 100 ms depolarizing pulse to 60 mV, which was employed to evoke an outward current. Cell-attached recordings were performed in voltage clamp with the current set around 0 pA. Data acquisition and analysis were performed using Clampex and Clampfit 10 (Axon Instruments, San Jose, CA, United States).

### Statistical Analysis

Data are expressed as mean ± SEM. One-way analysis of variance (ANOVA) was employed. In the presence of statistical significance, post hoc Bonferroni or S–N–K multiple comparisons were applied. Independent samples Student’s *t*-test was also used. All statistical tests were conducted using the SPSS 19.0 (IBM, Armonk, NY, United States) software package. Experimenters were blinded during the experiment and quantitative analysis was conducted to avoid biases. *p* < 0.05 was considered statistically significant.

## Results

### Adult Colorectal Distension Precipitates the Visceral Hypersensitivity in Rats Undergoing Neonatal Colorectal Distension

**FIGURE 1:**
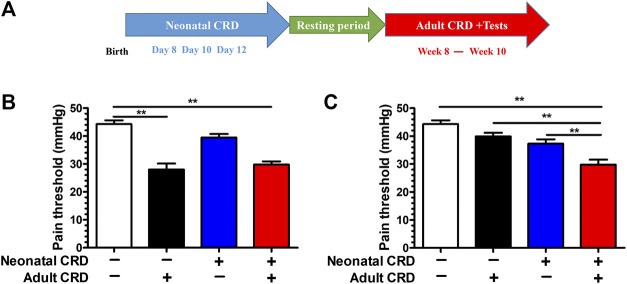
Adult CRD precipitated the visceral hypersensitivity in rats experiencing neonatal CRD. **(A)** Rats undergoing neonatal CRDs on postnatal days 8, 10, and 12, and adult CRD in week 8. The behavioral testing was conducted in weeks 8–10. **(B)** Pain threshold was measured immediately after adult CRD (n = 6 rats each, F_3,20_ = 25.83, *p* < 0.001). **(C)** Pain threshold was measured 2 h after adult CRD (n = 6 rats each, F_3,20_ = 16.34, *p* < 0.001). Significance was assessed by ordinary one-way ANOVA with post hoc comparisons between groups. The data are expressed as the mean ± SEM. **p < 0.01.

To evaluate the effect of adverse early stress on visceral pain sensitivity in rats, we adopted the paradigm of neonatal CRD. Rats were subjected to neonatal CRDs on postnatal days 8, 10, and 12, and adult CRD in week 8. The behavioral test was performed in weeks 8–10, which revealed evident decrease of pain threshold in rat groups (adult CRD; neonatal + adult CRD) (*p* < 0.01). (Figures 1A,B). Intriguingly, 2 h after adult CRD procedure, the pain threshold returned to normal in the adult CRD group, whereas the pain threshold remained significantly lower in the neonatal + adult CRD group (Figure 1C), suggesting that rats experiencing neonatal CRD presented with underlying visceral hypersensitivity, which can be revived and amplified by subsequent CRD in adulthood.

### Membrane SK2 Channel Protein and IAHP were Decreased in Rats Experiencing Neonatal Colorectal Distension

**FIGURE 2:**
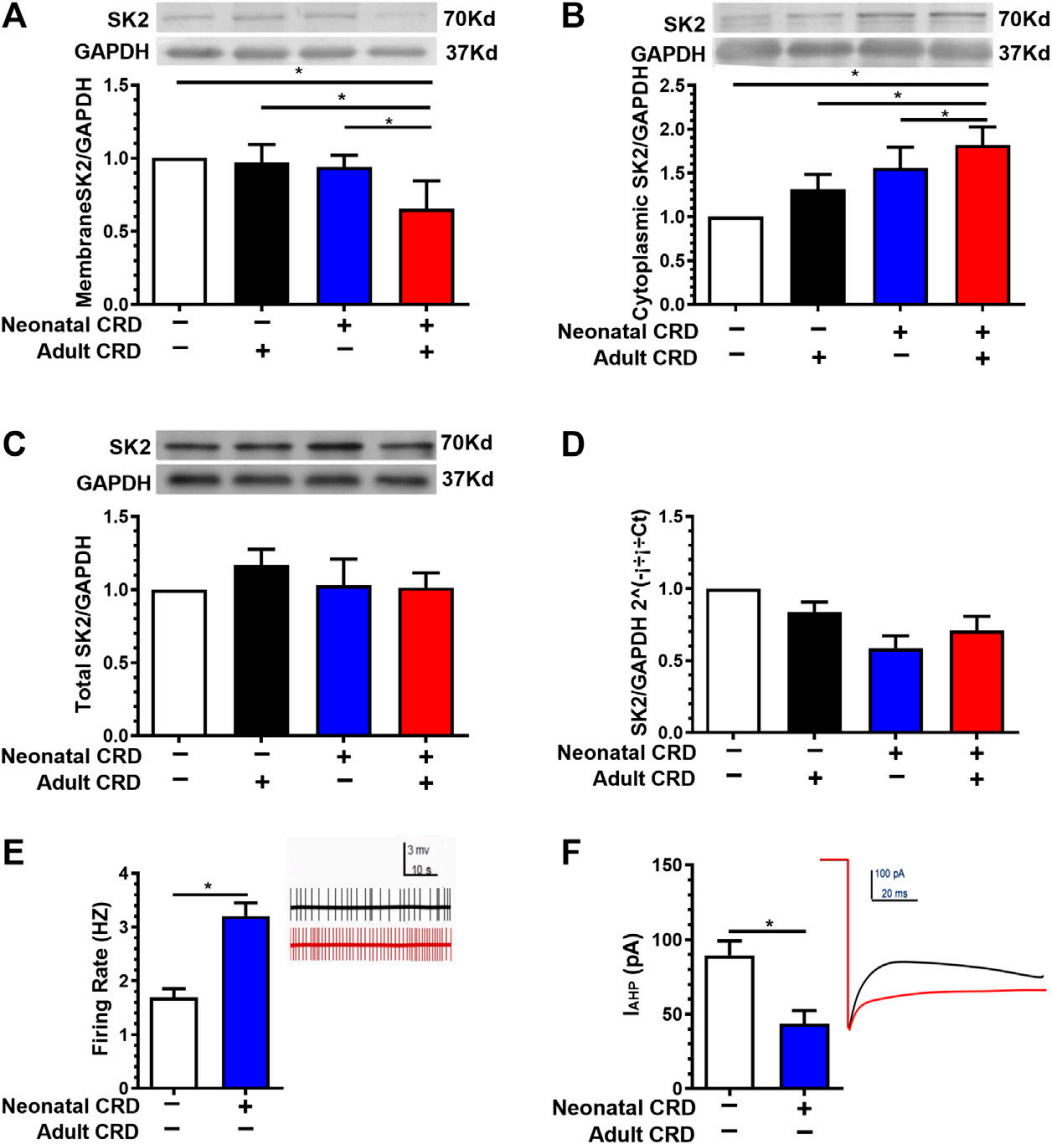
Membrane SK2 channel protein and IAHP were decreased in rats undergoing neonatal CRD. **(A)** Western blotting data revealed that the PVN SK2 channel protein in membrane fraction was decreased in rats undergoing neonatal and adult CRD vs. rats undergoing adult CRD alone (n = 6 rats each, F_3,20_ = 17.25, *p* < 0.0001). **(B)** The PVN SK2 channel protein in the cytoplasmic fraction was increased in rats undergoing neonatal and adult CRD vs. rats receiving adult CRD alone (n = 6 rats per group, F_3,20_ = 3.861, *p* = 0.0250). **(C,D)** Western blotting and qRT-PCR data showed insignificant between-group difference in total PVN SK2 protein (n = 6 rats each, F_3,20_ = 0.4638, *p* = 0.7108 in **C**; n = 6 rats each, F_3,20_ = 1.022, *p* = 0.4039 in **D)**. **(E,F)** Rats undergoing neonatal CRD presented with an increase in CRF neurons firing rates vs. the naïve rats (*p* < 0.05, n = 10 neurons each); consistently, rats undergoing neonatal CRD presented with low IAHP vs. rats without neonatal CRD (n = 13 cells each, t_24_ = 5.112, *p* < 0.0001 in E; n = 9 cells each, t_16_ = 6.913, *p* < 0.0001 in **F)**. Significance was assessed by ordinary one-way ANOVA with post hoc comparisons between groups in A, B, C, D, two-tailed unpaired Student’s *t*-test in E, **F**. The data are expressed as the mean ± SEM. *p < 0.05.

To address the physiological basis for visceral hypersensitivity in neonatal CRD rats, we next evaluated the SK2 expression and electrophysiological properties. Western blotting data revealed that the expression of PVN SK2 channel protein in membrane fraction was decreased in rats subjected to neonatal CRD (Figure 2A). Conversely, the PVN SK2 channel protein level in the cytoplasmic fraction was increased in rats undergoing neonatal CRD (Figure 2B). Moreover, Western blotting and qRT-PCR data showed insignificant between-group difference in total PVN SK2 protein (Figures 2C,D). Rats with neonatal CRD exhibited an increase in spontaneous neuronal firing rates vs. the naïve rats (Figure 2E). Consistently, rats with neonatal CRD presented with lower IAHP compared to rats without neonatal CRD (Figure 2F). These findings suggested that neonatal CRD contributed to the internalization of SK2 channels.

### Cell Membrane Protein Transport Inhibitor Dynasore Increased the Expression of Membrane SK2 Channel Protein and Alleviated Visceral Hypersensitivity

**FIGURE 3:**
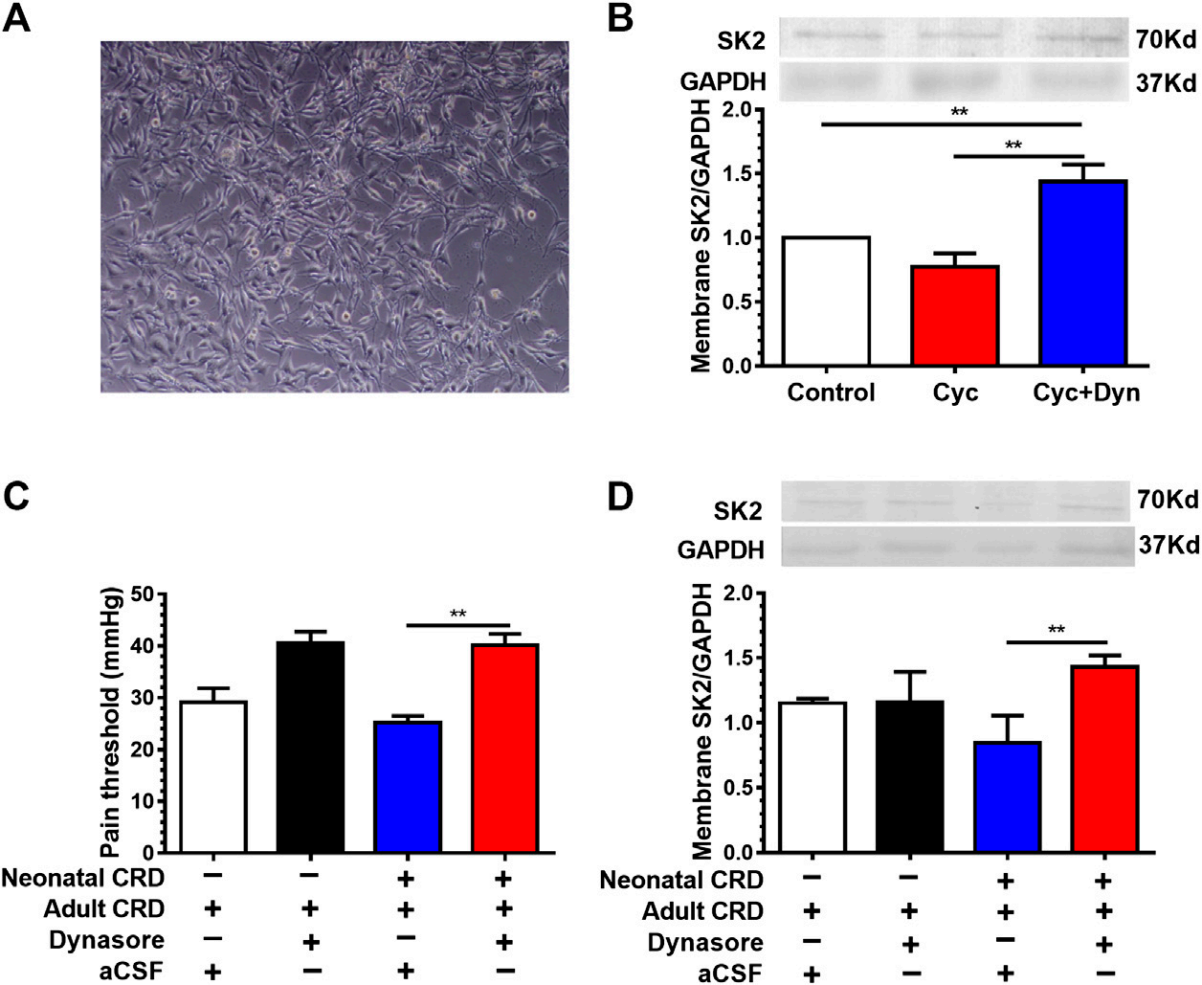
Cell membrane protein transport inhibitor dynasore increased the expression of membrane SK2 channel protein and alleviated visceral hypersensitivity. **(A)** Representative images of cultured HT22 hippocampal neurons *in vitro*. **(B)** Effect of dynasore (Dyn) on membrane SK2 channel protein expression *in vitro*. Western blotting results showed that the Dyn significantly increased the expression of membrane SK2 channel protein in HT22 hippocampal neurons (n = 6 rats each, F_2,15_ = 52.91, *p* < 0.0001). **(C,D)** Effect of intra-PVN injection of Dyn on pain threshold and membrane SK2 channel protein expression in neonatal CRD rats. Dyn precluded the decrease of membrane SK2 channel protein as well as the pain threshold in neonatal CRD rats (n = 6 rats each, F_3,20_ = 12.62, *p* < 0.0001 in **C**; n = 6 rats each, F_3,20_ = 3.637, *p* = 0.0305 in **D)**. Significance was assessed by ordinary one-way ANOVA with post hoc comparisons between groups. The data are expressed as the mean ± SEM. **p < 0.01.

To address the contribution of member SK2 channel protein to visceral hypersensitivity, we next adopted cell membrane protein transport inhibitor dynasore (Dyn). We initially tested the effect of Dyn (80 μmol/L) on membrane SK2 channel protein at the cellular level. Given the effect of intracellular SK2 protein synthesis during inhibition of protein transport, we employed the protein synthesis inhibitor cycloheximide (Cyc, 20 μg/ml) to antagonize the synthetic effect. Western blotting results revealed that the Dyn significantly increased the expression of membrane SK2 channel protein in cultured HT22 hippocampal neurons *in vitro* (Figures 3A,B), indicative of its effective inhibition of membrane SK2 channel protein transfer from the membrane into the cytoplasm.

To verify the effect of Dyn on visceral hypersensitivity in neonatal CRD rats, Dyn was administered by intra-PVN injection 30 min before behavioral testing. We identified that Dyn precluded the decrease of membrane SK2 channel protein as well as the pain threshold in rats (Figures 3C,D), suggesting that downregulation of membrane SK2 channel protein precipitated visceral hypersensitivity in neonatal CRD rats.

### SK2 Channel Activator 1-EBIO Decreased the CRF Neuronal Firing Rates and Alleviated Visceral Hypersensitivity

**FIGURE 4:**
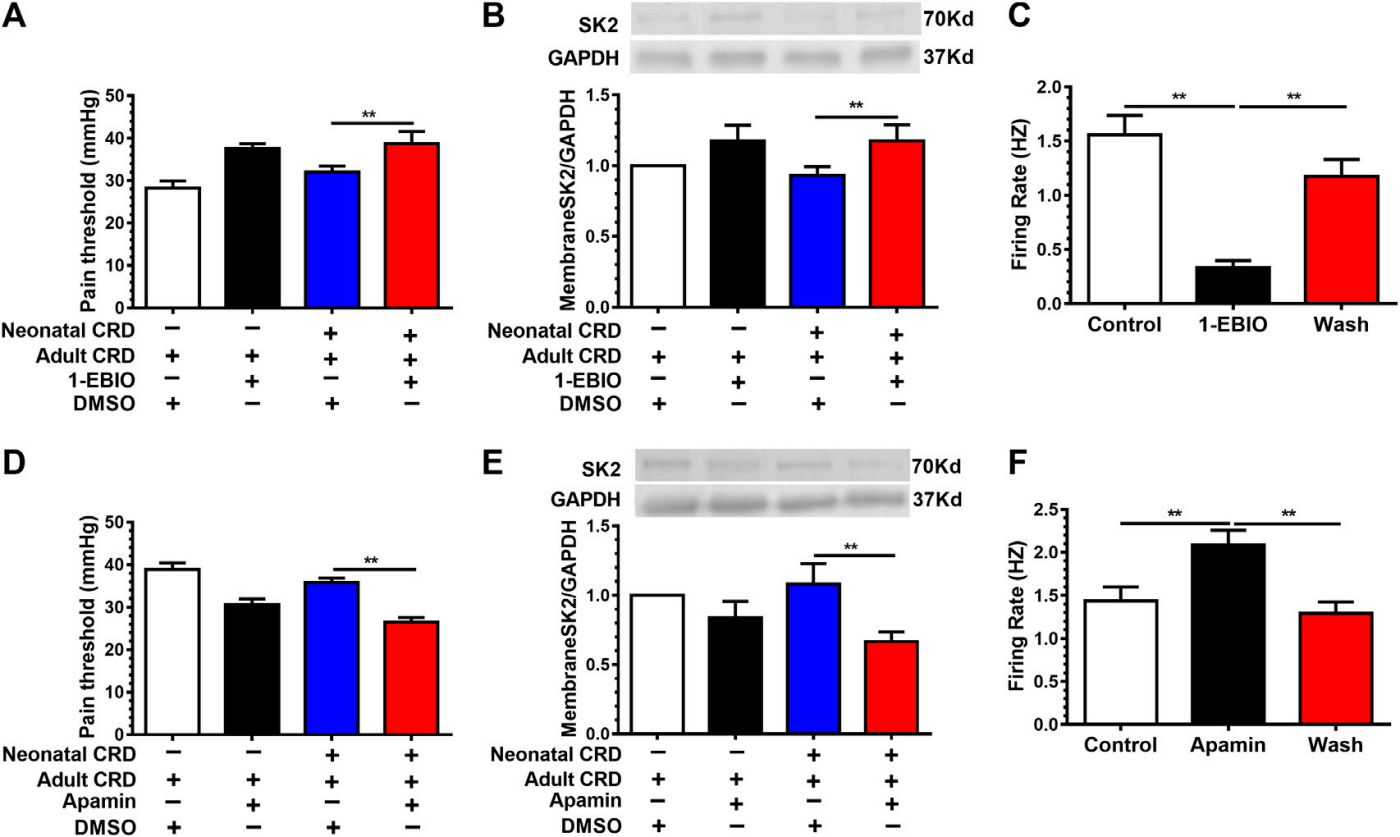
SK2 channel activator 1-EBIO decreased the neuronal firing rates and alleviated visceral pain. Rats receiving intra-PVN injection of 1-EBIO (10 μg/0.3 μL) 30 min before behavioral tests. 1-EBIO prevented the decrease of **(A)** the pain threshold; **(B)** the membrane SK2 channel protein in rats undergoing neonatal CRD (n = 6 rats each, F_3,20_ = 27.4, *p* < 0.0001 in **A**; n = 6 rats each, F_3,20_ = 5.387, *p* = 0.0070 in **B)**. **(C)** SK channel activator 1-EBIO (100 mM) reversed the increase of the CRF neurons firing rates (n = 10 cells each, F_2,27_ = 41.91, *p* < 0.0001). SK2 channel blocker apamin (6.25 pmol/0.3 μL) decreased **(D)** the pain threshold; **(E)** the expression of membrane SK2 channel protein in rats undergoing neonatal and adult CRD (n = 7 rats each, F_3,24_ = 19.97, *p* < 0.0001 in **D**; n = 6 rats each, F_3,20_ = 8.901, *p* = 0.0006 in **E)**. **(F)** SK channel blocker apamin increased the CRF neurons firing rates (n = 9 cells each, F_2,24_ = 7.95, *p* = 0.0022). Significance was assessed by ordinary one-way ANOVA with post hoc comparisons between groups. The data are expressed as the mean ± SEM. **p < 0.01.

To authenticate whether activation of SK2 channel mitigates visceral hypersensitivity in neonatal CRD rats, 1-EBIO (10 μg/0.3 μL) was injected (intra-PVN). 30 min after injection, 1-EBIO precluded the decrease of pain threshold. Notably, 1-EBIO even rescued the downregulation of membrane SK2 channel protein in neonatal CRD rats (Figures 4A,B). In addition, SK channel activator 1-EBIO (100 mM) reversed the increase of spontaneous firing rates of CRF neurons (Figure 4C). These results revealed that 1-EBIO could upregulate the activity of SK2 channel, thereby reducing the CRF neuronal excitability in PVN as well as alleviating visceral hypersensitivity.

To further explore the specific contribution of SK2 inhibition to visceral hypersensitivity in neonatal CRD rats, SK2 channel selective blocker apamin (6.25 pmol/0.3 μL) was administered (intra-PVN). We identified further reduction of membrane SK2 channel protein expression and the pain threshold in neonatal CRD rats (Figures 4D,E). Moreover, apamin increased the firing rates of CRF neurons (Figure 4F). These results indicated that SK2 inhibition precipitated visceral hypersensitivity in neonatal CRD rats.

Together, these findings demonstrated the potential of SK2 activator as an alternative agent for visceral pain.

### PKA Activation Facilitated the Transfer of SK2 Channel Protein from the Cell Membrane into the Cytoplasm

**FIGURE 5:**
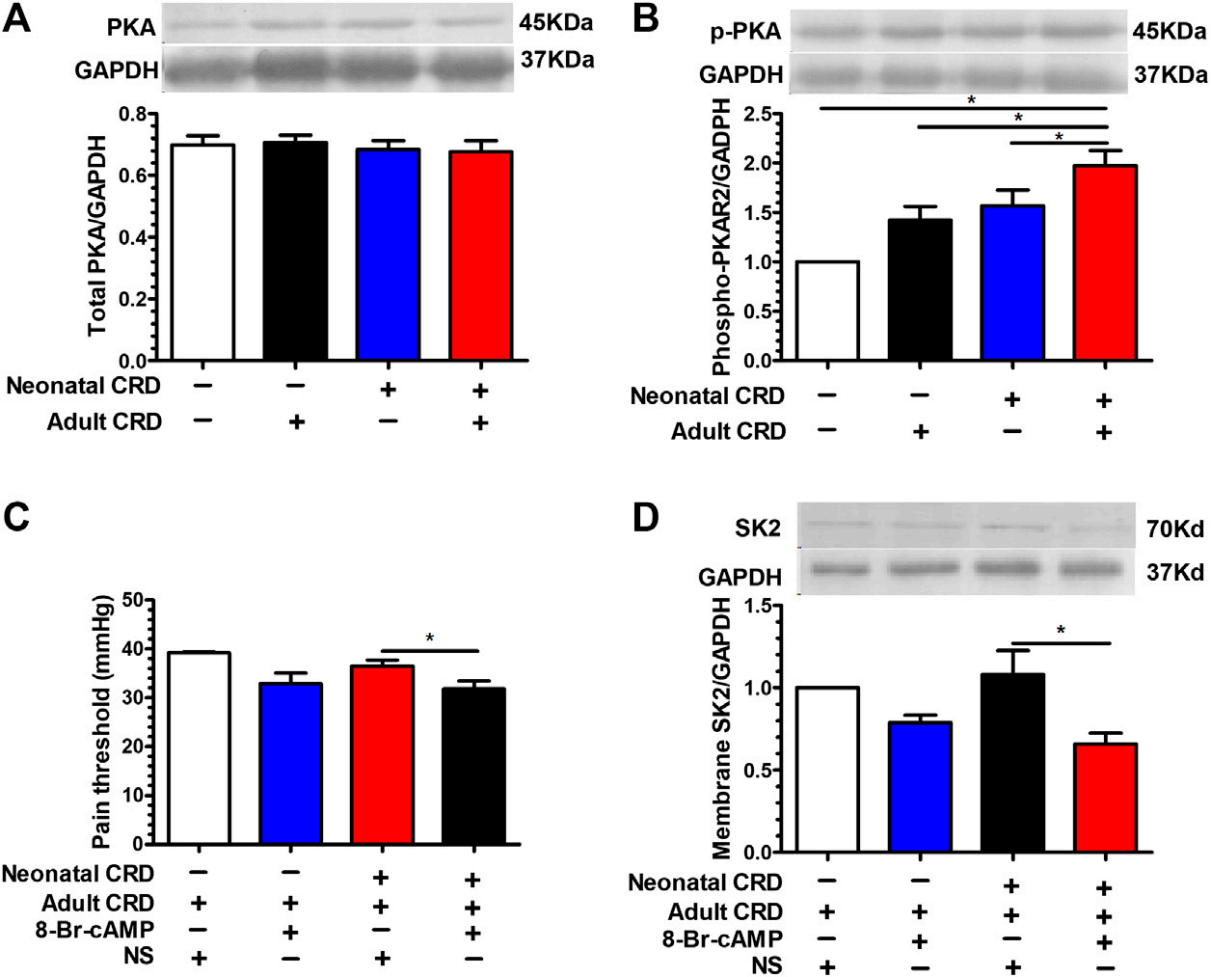
PKA activation facilitated the transfer of SK2 channel protein from the cell membrane into the cytoplasm. **(A)** There was insignificant change in the total PKA expression (n = 6 rats each, F_3,20_ = 0.3017, *p* = 0.8238). **(B)** The expression of phosphorylated PKA in PVN was significantly increased in rats undergoing neonatal CRD vs. other groups (n = 6 rats each, F_3,20_ = 30.44, *p* < 0.0001). Intra-PVN administration of PKA agonist 8-Br-cAMP (100 mM, 0.1 μl) decreased **(C)** pain threshold as well as **(D)** membrane SK2 channel protein in Neonatal CRD group (n = 6 rats each, F_3,20_ = 19.24, *p* < 0.0001 in **C**; n = 6 rats each, F_3,20_ = 15.33, *p* < 0.0001 in **D)**. Significance was assessed by ordinary one-way ANOVA with post hoc comparisons between groups. The data are expressed as the mean ± SEM. *p < 0.05.

On the grounds that SK2 channel regulates the pain threshold, we proceeded to explore the physiological basis for SK2 channel protein transfer from membrane into cytoplasm in neonatal CRD rats. Prior studies revealed that direct PKA phosphorylation of SK2 alters the distribution of SK2 channel proteins. Nevertheless, puzzles remain with respect to the potential correlations between PKA responses in CRF neurons and distribution of SK2 channel proteins as well as precipitation of behavioral abnormalities, such as visceral hypersensitivity in neonatal CRD rats. Intriguingly, no marked variations in the total PKA expression were evidenced (Figure 5A); whereas the expression of phosphorylated PKA in PVN was significantly increased in neonatal rats (Figure 5B). Intra-PVN administration of PKA agonist 8-Br-cAMP (100 mM, 0.1 μl) decreased membrane SK2 channel protein levels as well as pain threshold in neonatal CRD rats (Figures 5C,D). The results revealed that PKA was implicated in membrane translocation of SK2 channel in neonatal CRD rats.

## Discussion

In the present study, we validated that neonatal CRD contributed to visceral hypersensitivity. As per the assessment of the expression of SK2 channel protein that exhibited transfer from membrane into cytoplasm, and combination with the electrophysiological profiles of SK2 channel in CRF neurons, we postulated that decreased membrane SK2 expression precipitated abnormal neuronal physiology and visceral hypersensitivity. We also identified the efficacy of an SK2 activator in mitigating visceral hypersensitivity in neonatal CRD rats, demonstrating the potential of this approach as an intervention for visceral pain. Moreover, we disclosed PKA was the primary culprit for SK2 distribution anomalies in neonatal rats, which may provide novel targets for diseases caused by abnormalities of SK2 distribution including visceral pain.

Visceral hypersensitivity is among the key pathophysiological features of IBS as well as other disorders with visceral pain, for which no efficient therapies are available, thus rendering significant adverse effect on the quality of life (Wouters et al., 2012; Chey et al., 2015). Despite the obscure mechanism underlying visceral hypersensitivity, adverse early life stress may potentially contribute to the precipitation (Al-Chaer et al., 2000; Zhou et al., 2010; Larauche et al., 2012). Early life stress can predispose the developmental track of neuroendocrine system in the CNS, particularly the HPA axis, and precipitate the susceptibility to subsequent stressors and development of visceral hypersensitivity in adulthood (Ji et al., 2018). The hypothalamic CRF neurons are implicated in the activation of stress-induced HPA axis (Kusek et al., 2013), further supported by our prior report that visceral hypersensitivity is related to an increase of CRF mRNA and protein expression levels as well as CRF neuronal activation in PVN (Zhang et al., 2016a). Thus, it is essential to explore the CRF neuronal molecular mechanism underlying visceral hypersensitivity in PVN.

Our prior initial study focused on the first key relay stations of various visceral primary afferent information, i.e. dorsal horn (DH), and we found that a decrease in the number and function of membrane SK2 channels increased neuronal excitability in the spinal DH, leading to increased sensitivity to noxious stimuli and subsequent visceral hypersensitivity, which indicated the important role of SK2 in the ascending pathway (Song et al., 2018). Since it has been clarified that SK2 is involved in the regulation of chronic visceral pain, we want to further disclose the role of SK2 in the higher level key relay stations of various visceral primary afferent information and the hub of visceral sensation, i.e. PVN, and study the underlying mechanisms for regulating SK2 dynamic change in chronic visceral pain in this paper.

Coincidentally, we found that neonatal CRD rats presented with a downregulation of the membrane SK2 channel protein in PVN, and the expression of the membrane SK2 channel protein was correlated with visceral hypersensitivity. Therefore, we hypothesized that SK2 channel in PVN is associated with visceral hypersensitivity. In addition, CRF neurons exhibited altered physiological properties, i.e. lower SK2 channel-mediated IAHP. Previous studies reported activation of SK2 channel is the prerequisite for the generation of medium afterhyperpolarization potential (mAHP) following single or multiple action potentials, leading to a decrease of the firing rate and inhibition of the excitatory of neurons (Pedarzani et al., 2005; Lu et al., 2009). Therefore, decreased SK2 channel-mediated IAHP may be attributed to the increased excitability of CRF neurons in PVN and aberrant modification of HPA axis in neonatal CRD rats with visceral hypersensitivity.

Given the contribution of the SK2 channel redistribution to visceral hypersensitivity, neonatal CRD rats should be well restored, provided SK2 is precluded from transference from the membrane into the cytoplasm. Indeed, this hypothesis was confirmed by the use of cell membrane protein transport inhibitor Dyn. This evidence further testifies the impact of abnormal SK2 distribution on visceral hypersensitivity, and may also render a therapeutic strategy for the diseases due to abnormal membrane protein internalization, including visceral pain. Our administration of SK2 channel activator 1-EBIO reversed the increase of neuronal firing rate in the CRF neurons in PVN and further relieved the visceral hypersensitivity in neonatal CRD rats, demonstrating the potential of this approach as an intervention for visceral pain.

Of note, membrane SK2 channel protein downregulation in neonatal CRD rats was not restricted to CRF neurons according to western blotting results. Despite our finding that SK2 channel activation in PVN mitigated the visceral hypersensitivity in neonatal CRD rats, whether SK2 channel activation in other neuronal populations can also serve as a therapeutic strategy for the treatment of neonatal CRD-induced visceral hypersensitivity awaits further exploration. Moreover, since 1-EBIO per se is not a selective SK2 activator, one cannot simply exclude the possibility of the involvement of other potassium channels in visceral hypersensitivity.

Nonetheless, the contributor of the downregulation of membrane SK2 channel protein in PVN remains unclear. Direct PKA phosphorylation of SK2 alters the distribution of SK2 channel proteins in the COS7 expression system, which may play a pivotal role in the regulation of the ImAHP and excitability of the nervous system (Ren et al., 2006; Abiraman et al., 2016). Activation of PKA leads to internalization of synaptic SK2 channel and enhances excitatory synaptic transmission and plasticity in amygdala and hippocampal neurons (Faber et al., 2008; Lin et al., 2008). Besides, activation of the cAMP-PKA signaling pathway in rat dorsal root ganglion and spinal cord contributes toward induction and maintenance of bone cancer pain (Zhu et al., 2014). Consistent with these reports, our present study confirmed that the PKA agonist 8-Br-cAMP increased the expression of phosphorylated PKA, which further downregulated the membrane SK2 channel protein and upregulated the cytoplasmic SK2 channel protein in PVN, facilitating the transfer of SK2 channel protein from the cell membrane into the cytoplasm, and reversely aggravated visceral hypersensitivity. A priori, CRD-induced visceral hypersensitivity decreased the membrane SK2 channel via the promoted PKA activity in PVN.

In this study, the redistribution of the SK2 channel in PVN participates in chronic visceral pain. The hypothalamic PVN integrates multiple afferents to precipitate autonomous output of pain and analgesia regulation (Wouters et al., 2012; Chen et al., 2014). SK channels, extensively distributed in the brain regions including PVN (Adelman et al., 2012; Deignan et al., 2012), modulate neuronal excitability, which further modulate neuropathic pain (Thompson et al., 2015). There is a wealth of evidence that the redistribution of the SK2 channel at the cell level is related to the phosphorylation of PKA in the membrane (Majekova et al., 2017; Authement et al., 2018). This study authenticated that visceral hypersensitivity is implicated in PKA-regulated membrane SK2 channel in PVN.

**FIGURE 6:**
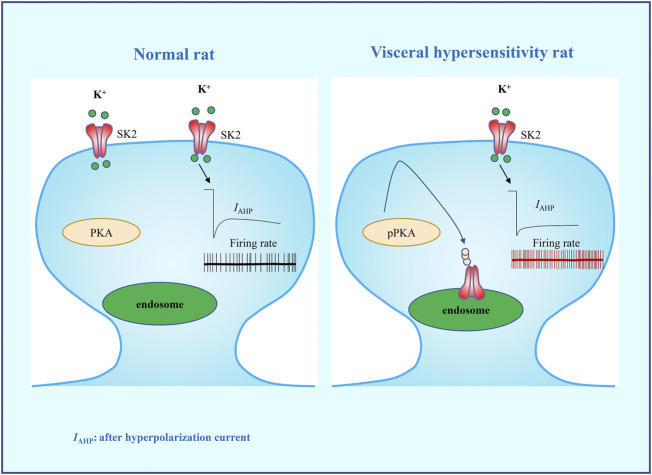
A schematic diagram summarizing the hypothesis that PKA-regulated membrane SK2 channel in PVN precipitates neonatal CRD-induced visceral hypersensitivity. Activation of PKA facilitates the internalization of SK2 channel and the decrease of IAHP, thereby increasing the firing rate of CRF neurons in PVN, which precipitates neonatal CRD-induced visceral hypersensitivity.

In conclusion, we evaluated the mechanism of the SK2 channels in PVN in the etiology of visceral pain. Our data confirmed that rats with visceral hypersensitivity present with a downregulation of membrane SK2 channel as well as functional inhibition in PVN, which may be related to PKA activation (Figure 6). Intra-PVN administration of SK2 channel activator 1-EBIO can reverse the visceral hypersensitivity. This study may provide an insight into the pathogenesis of visceral pain and pave a novel avenue for the development of therapeutic regimens for visceral pain.

## Data Availability Statement

The original contributions presented in the study are included in the article/Supplementary Material, further inquiries can be directed to the corresponding authors.

## Ethics Statement

The animal study was reviewed and approved by Institutional Animal Care and Use Committee at Xuzhou Medical University.

## Author Contributions

Participation in research design: Y‐MZ and RH. Experimentation: N‐NJ, LD, YW and KW. Data analysis: N‐NJ and LD. Manuscript composition or revision: N‐NJ, Z‐YC, RH and Y‐MZ. All authors contributed to the article and approved the submitted version.

## Funding

This research was funded by the National Natural Science Foundation of China (Grant Numbers 82071228; 81771203; 81772065); Key Subject of Colleges and Universities Natural Science Foundation of Jiangsu Province (Grant Number 19KJA110001 to YZ) and sponsored by Qing Lan Project; Postgraduate Research & Practice Innovation Program of Jiangsu Province (KYCX20_2450).

## Conflict of Interest

The authors declare that the research was conducted in the absence of any commercial or financial relationships that could be construed as a potential conflict of interest.
